# Research progress on the regulation of autophagy in cardiovascular diseases by chemokines

**DOI:** 10.1515/biol-2022-1026

**Published:** 2025-06-17

**Authors:** Jingfeng Ma, Xiaotong Tan, Juling Feng, Zhihui Li, Shuo Tan, Boling Li, Lei Zhao

**Affiliations:** The First Affiliated Hospital, Department of Gastrointestinal Surgery, Hengyang Medical School, University of South China, Hengyang, Hunan, 421001, China; Research Lab of Translational Medicine, School of Basic Medical Sciences, Hengyang Medical School, University of South China, Hengyang, Hunan, 421001, China

**Keywords:** chemokines, autophagy, cardiovascular diseases, CXCR4/CXCL12, CXCL16/CXCR6, CX3CL1

## Abstract

Cardiovascular diseases (CVD) are the leading cause of death worldwide. Chemokines are a class of proteins that possess characteristics of both chemoattractants and cytokines. They play a pivotal role in CVD by regulating the recruitment of immune cells and suppressing inflammatory responses. These proteins are crucial for maintaining cardiac function and for managing myocardial cell damage under various stress conditions. Autophagy, a vital intracellular degradation mechanism, is essential for clearing misfolded proteins and damaged organelles, thus promoting cell survival during starvation and other stress conditions. A substantial body of research indicates that chemokines can modulate the development of CVD by influencing the autophagy process. Research has shown that targeting pathways such as CXCR4 and CXCL12, defective CXCL16/CXCR6, and inhibiting CX3CL1 can regulate autophagy and impact CVD. The protective role of chemokines in CVD through the modulation of autophagy may offer new perspectives for treatment.

## 1 Introduction

Cardiovascular diseases (CVD) continue to be a major cause of death globally, with atherosclerotic CVD accounting for the majority of cardiovascular mortality [1]. Extensive research has revealed a close connection between chemokines and CVD. Chemokines can influence CVD through regulation of the recruitment of immune cells to migrate to the site of injury, thereby suppressing inflammation, carrying out immune defense, tissue reconstruction, and adjusting pathological responses in disease states. In the global response to CVD, primary prevention strategies are also particularly important. Smoking, unhealthy eating habits, a lack of physical exercise, excessive alcohol consumption, and hypertension are considered the main preventable risk factors for CVD [2,3].

Autophagy is a critical intracellular degradation mechanism that primarily involves the clearance of proteins and damaged organelles due to aging or misfolding, thus facilitating cell survival under starvation and stress conditions [4]. Under both homeostatic and stress conditions, autophagy is crucial in cardiomyocytes and has emerged as a potential therapeutic target for preventing heart disease. Disruptions in myocardial cell autophagy, particularly its impairment, are central to the pathogenesis of heart failure, hypertrophic cardiomyopathy, dilated cardiomyopathy, cardiac aging, diabetic cardiomyopathy, and ischemia/reperfusion (I/R) injury [5,6].

The upstream signals of autophagy are transmitted mainly through mTOR-dependent and mTOR-independent pathways, including the AMPK, PI3K, Ras-MAPK, p53, and PTEN pathways, and through endoplasmic reticulum stress. Recent studies have shown that chemokines can influence CVD pathogenesis and progression by affecting autophagy, such as by blocking CXCR4 combined with its specific receptor CXCL12 through the PI3K/AKT/mTOR pathway to increase macrophage autophagy, alleviate atherosclerosis, and improve myocardial structure. Defective CXCL16/CXCR6 may protect the heart by inhibiting monocyte infiltration and IFN-γ production, thereby inducing cardiac autophagy. Inhibition of CX3CL1 can reverse the therapeutic effects of NPAS2 on myocardial injury [7,8,9]. This review summarizes recent literature on the impact of chemokines on CVD through autophagy, aiming to provide new insights into the mechanisms of chemokines in CVD and identify new drug targets for treatment.

## 2 Chemokines

Chemokines are a class of cytokines with a molecular weight of approximately 8–12 kDa [10] that play a role in coordinating immune responses during acute and chronic tissue damage in mammalian organs [11]. They are characterized by four conserved cysteine residues that form two disulfide bonds, with the first and third as well as the second and fourth cysteines paired [12]. A single chemokine can bind to multiple chemokine receptors, and a single chemokine receptor can have multiple ligands. To date, 47 chemokines and 20 chemokine receptors have been identified in humans [7]. Chemokines can affect monocyte survival, leukocyte activation, foam cell development, smooth muscle cell proliferation, and angiogenesis. Thus, chemokines induce endothelial dysfunction, atherosclerosis formation, and cardiac hypertrophy by mediating leukocyte chemotaxis, vascular cell migration, and proliferation. Changes in chemokines and their receptors play a significant role in the pathophysiology of CVD [10].

### 2.1 Classification of chemokine ligands

Chemokines are classified into CC, CXC, CX3C, and C families on the basis of the position of the cysteine residues [13]. Both the CC and CXC families contain four cysteines. The CC family includes 28 members, ranging from CCL1 to CCL28. The CXC family includes 17 members, ranging from CXCL1 to CXCL17.

#### 2.1.1 CCL chemokine ligands

The CC family is the largest group of chemokines. CCL1 is primarily a T-cell product that is secreted by macrophages, dendritic cells, mast cells, and tumor cells. It is an effective chemoattractant for monocytes and lymphocytes and is expressed in endothelial cells, macrophages, and atherosclerotic plaques, stimulating vascular smooth muscle cell migration and endothelial cell activation [14,15]. CCL2, also known as monocyte chemoattractant protein-1, facilitates monocyte attraction, influences monocyte and lymphocyte phenotypes, and contributes to fibrous tissue accumulation, blood vessel formation, and atherosclerotic plaque development. It affects the migration of leukocytes to the arterial wall by controlling the movement of inflammatory cells and is significantly induced in the infarcted myocardium [16,17]. CCL3 is derived primarily from macrophages and induces chemotaxis of different leukocyte subsets, including monocytes/macrophages and T lymphocytes, through CCR1, CCR4, or CCR5. Activated platelets, neutrophils, and mast cells can release this chemokine, and neutrophils stimulated by tumor necrosis factor-alpha respond to CCL3, contributing to atherosclerosis overall by upregulating the integrins CD11b and CD18 [18,19,20]. CCL5 is one of the most widely used chemokines and acts on three chemokine receptors, namely, CCR1, CCR3, and CCR5. It is highly induced in many cells and is stored in large amounts in platelet alpha granules and cytoplasmic granules of memory or effector CD8+ T cells. For example, CCL5 facilitates the recruitment and penetration of immune cells into atherosclerotic plaques, thus playing a pivotal role in orchestrating the homing of T cells in atherosclerotic lesions [20,21]. CCL14 and CCL15 are present in high concentrations in serum, have an extended N-terminus, and are converted into high-affinity ligands through N-terminal cleavage. CCL23 also has a long N-terminus and is closely related to CCL15. CCL17 is expressed mainly by a subset of myeloid dendritic cells and promotes atherosclerosis by mediating T-cell chemotactic activity through its receptor CCR4 [22,23]. Increased levels of CCL19 and CCL21 in atherosclerosis can lead to changes in T-cell and macrophage responses by regulating dysfunction, thus reducing plaque stability and contributing to atherosclerosis development [24]. CCL20 is produced by cells associated with inflammation and autoimmune responses, including endothelial cells, neutrophils, NK cells, TH17 cells, and B cells. CCL20 selectively acts on its receptor CCR6, chemoattracting dendritic cells, effector/memory T lymphocytes, and naive B cells, with significantly elevated levels in atherosclerotic lesions [25,26]. CCL24 is primarily produced by activated immune cells (especially M2-type macrophages) and epithelial cells. It plays an important role in various inflammatory responses, fibrotic processes, and vascular-related biological activities. In addition, elevated levels of CCL24 are closely associated with increased risk of CVD attacks [27,28]. Most members of the CC family are involved in the development of CVD, particularly the pathogenesis of atherosclerosis. Their role in regulating immune cell recruitment, inflammation, and plaque formation highlights their potential as targets for therapeutic intervention and prevention strategies for CVD.

#### 2.1.2 CXCL chemokine ligands

CXC chemokines are the second-largest group of chemokines and are characterized by a nonconserved single amino acid between the N-terminal cysteine residues. CXCL1 is found primarily in cells such as neutrophils, macrophages, and epithelial cells. It promotes the recruitment of neutrophils and monocytes/macrophages to the injured heart and arterial walls through its receptor CXCR2, inducing myocardial infarction, I/R injury, atherosclerosis, and hypertension [29]. CXCL1 is also involved in the fibrosis of various organs, such as cardiac and liver fibrosis. Endothelial cells release CXCL1 when induced by oxLDL, and high levels of CXCL1 can lead to leukocyte recruitment and migration to the carotid bifurcation, accelerating the progression of atherosclerosis [30]. CXCL4 is a chemokine that is stored in large amounts in platelets; during injury and atherosclerosis, it affects the function of vascular smooth muscle cells by regulating proliferation, migration, gene expression, and cytokine release through vascular wall transport. CXCL4L1 is a nonallelic variant of CXCL4 [31]. CXCL4, CXCL4L1, CXCL7, and CCRL5 comprise a group of chemokines that can be highly concentrated in platelet alpha granules and rapidly released upon platelet activation, thus playing important roles as inflammatory mediators of vascular injury, with CXCL4 being one of the oldest members of the chemokine superfamily [32,33]. CXCL8, named neutrophil-activating factor, monocyte-derived neutrophil-activating peptide, and monocyte-derived neutrophil chemotactic factor, is produced by various cell types. CXCL10, an inducer of smooth muscle cell migration, also known as interferon-gamma-induced protein 10, increases vascular wall endothelial permeability and is highly induced in endothelial cells, fibroblasts, keratinocytes, monocytes, T lymphocytes, and macrophages by interferon [10,34]. CXCL8 and CXCL10 increase their expression in atherosclerotic lesions by mediating chemotaxis and mitogenic effects on neutrophils, T cells, and vascular smooth muscle cells [32]. CXCL12, also known as stromal cell-derived factor 1α, plays a key role in the migration and differentiation of leukocytes, hematopoietic stem cells, and progenitor cells, and has an important function in the repair process of ischemic tissue. CXCL12 derived from endothelial cells affects the progression of atherosclerosis, and the source of its release has a crucial impact on its role in vascular inflammation [32,35]. CXCL16 belongs to the α-chemokine subfamily and contains a mucin stalk, transmembrane, and cytoplasmic domains. It exists in two forms, membrane-bound and soluble, and is expressed on activated T cells and natural killer T cells. Soluble CXCL16 has a proatherosclerotic effect [36,37]. The majority of CXC family members influence CVD onset and progression, particularly myocardial infarction, I/R injury, atherosclerosis, and hypertension, and hold potential as targets for CVD treatment and prevention. They are also implicated in fibrotic processes affecting various organs, including cardiac fibrosis and liver fibrosis. They hold potential value for the treatment and prevention of CVD; however, specific therapeutic and preventive approaches require further research and support from clinical observations.

#### 2.1.3 CX3CL chemokine ligands

CX3CL1 is the only known membrane-bound chemokine with both chemoattractant and adhesive functions [38]. CX3CL1 is expressed on activated endothelial cells, smooth muscle cells, macrophages, and platelets and is involved in the development of inflammatory pathologies such as atherosclerosis. It mediates chemotaxis through the CX3CR1 receptor and can act as a medium for smooth muscle cell migration [10]. On the surfaces of endothelial and epithelial cells, membrane-bound CX3CL1 is involved mainly in adhesion and plays a role in capturing CX3CR1-positive neutrophils, whereas the soluble form of CX3CL1 acts primarily as a chemokine, affecting the chemotaxis of monocytes, natural killer cells, dendritic cells, and T cells [20,38].

### 2.2 Classification of chemokine receptors

Chemokine receptors are class A G protein-coupled receptors (GPCRs) associated with heterotrimeric Gai class G proteins. The primary chemokine ligands are divided into four subfamilies, namely, CCR, CXCR, CX3CR, and XCR, with 10 CC chemokine receptors, 6 CXC chemokine receptors, 1 CX3C chemokine receptor, and 1 XC chemokine receptor [39]. Most chemokine receptors can interact with many different ligands within the same class, and ligands and receptors may also form heterodimers.

#### 2.2.1 CC chemokine receptors

CCR1 is expressed on monocytes, neutrophils, and T cells, and is involved in various inflammatory responses, including ischemia-reperfusion injury and immune reactions. It binds several chemokine ligands, including CCL5, CCL3, and CCL4, and its role in several inflammatory diseases, such as ischemia-reperfusion injury, is well characterized [39,40]. CCR2, a GPCR, mediates the recruitment of monocytes and is an effective promoter during the inflammatory phase of neutrophil recruitment. The CCL2-CCR2 axis, formed by the binding of CCR2 with CCL2, is associated with the progression of various diseases, such as atherosclerosis, diabetes, and cancer. Therefore, the CCL2-CCR2 axis is considered a potential therapeutic target for these diseases [41,42]. CCR5 is expressed mainly on activated T cells and monocytes/macrophages and mediates the activity of the chemokines CCL3, CCL4, and CCL5. It has an atherogenic phenotype, and the expression of CCR5 is significantly increased during inflammatory infiltration in various diseases, indicating that this receptor plays a role in transporting cells to inflammatory sites [21,43]. CCR1, CCR2, and CCR5 play major roles in the firm adhesion of leukocytes under ischemic conditions, helping to recruit leukocytes to reperfused tissues. CCR7 and its two ligands, CCL19 and CCL21, are crucial for the homing of lymphocytes and dendritic cells to secondary lymphoid tissues. Dendritic cells equipped with CCR7 can accumulate in the T-cell zone of the lymph node, thereby initiating the process of antigen presentation and eliciting T-cell reactions. Abnormal expression of CCR7 in dendritic cells can cause a series of inflammatory diseases due to disordered dendritic cell-derived transport [44]. CCR7 plays a role in regulating the migration of dendritic cell exosomes, thereby improving cardiac function after myocardial infarction [45]. Most CCR8+ cells are lymphocytes, especially CD4+ αβ T cells, as well as a few γδ T cells and NK cells. Disruption of the binding of the CCL1-CCR8 axis can impair the recruitment and function of vascular Treg cells, promoting atherosclerosis [14,46].

#### 2.2.2 CXC chemokine receptors

CXCR1 and CXCR2 are expressed across multiple cell types, such as white blood cells, fibroblasts, endothelial cells, and smooth muscle cells. Their main role is to stimulate the directional movement of leukocytes, especially neutrophils, which is essential for guiding T and NK cells to areas of inflammation. They are also involved in the mobilization of neutrophils and the early formation of atherosclerotic plaques. CXCR2 may mediate leukocyte recruitment that leads to cell death during ischemia-reperfusion, as well as direct cell protection that leads to survival, with the recruitment of damaging inflammatory cells being stronger than the direct protective effect of resident cells on the myocardium [47]. CXCR3 is a transmembrane G protein-coupled receptor that selectively binds to its functional ligands CXCL9, CXCL10, and CXCL11. Predominantly expressed on immune cells, CXCR3 is expressed on activated T lymphocytes, natural killer cells, and monocytes/macrophages. Therefore, it plays a crucial role in immune processes, such as regulating the direction of effector cells to the site of infection and clearing pathogens [48]. Furthermore, it is also expressed in a variety of cell types within the cardiovascular system and is associated with several systemic diseases, such as atherosclerosis, chronic Chagas cardiomyopathy, and hypertension [49]. CXCR4 is a key immune response regulatory factor that is mediated by macrophages. The infiltration of CXCR4+ macrophages in the heart exacerbates hypertension-induced diastolic dysfunction by promoting the production of proinflammatory cytokines [50]. CXCR4 is expressed in stem cells, peripheral blood leukocytes, endothelial cells, smooth muscle cells, and myocardial cells. Together with its ligand CXCL12, it coordinates rapid vascular reconstruction of damaged, ischemic, and regenerative tissues and plays a key role in ischemic responses. CXCR7 has a tenfold greater affinity for CXCL12 than CXCR4 and is generally considered a negative regulatory factor for the expression and function of CXCL12. Upon binding of their ligand CXCL12, CXCR4 and CXCR7 play significant roles in cardiovascular development. Additionally, CXCR7 has crucial functions in the morphogenesis and remodeling of cardiac valves following myocardial infarction [51]. CXCR6 plays a proatherosclerotic role in atherosclerosis and regulates the recruitment of proinflammatory IL-17A-producing T cells to atherosclerotic aortas [36].

#### 2.2.3 CX3C chemokine receptor

CX3CR1 is a G protein-coupled receptor with seven transmembrane domains that can guide cells to closely adhere to the fixed form of CX3CL1 under *in vitro* flow conditions. Vascular smooth muscle cells express the CX3CR1 receptor and play a role in the formation of platelet‒monocyte complexes [20,38] (Table 1).

**Table 1:** Chemokines potentially associated with CVD

Chemokine	Expression location/source	Main function
**Classification of chemokine ligands**		
CCL1	Pancreatic duct epithelium, peribiliary glands, vascular endothelial cells, CCR8+ lymphocytes	—
CCL2	Monocytes, lymphocytes, platelets, neutrophils, mast cells	Regulates monocyte and lymphocyte phenotypes, promotes atherosclerosis formation [16,17]
CCL3	Macrophages, activated platelets, neutrophils, mast cells	Induces chemotaxis of different leukocyte subsets through CCR1, CCR4, or CCR5, leading to atherosclerosis [18,19,20]
CCL5	Platelet alpha granules, cytoplasmic granules of CD8+ T cells, various cells	Mediates immune cell recruitment and infiltration into atherosclerotic plaques [20,21]
CCL14	High concentration in serum	—
CCL15	High concentration in serum	—
CCL17	Subset of myeloid dendritic cells	Mediates T-cell chemotactic activity through CCR4, promotes atherosclerosis [22,23]
CCL19	—	Changes in T-cell and macrophage responses due to dysfunction, reducing plaque stability, contributes to atherosclerosis [24]
CCL20	Endothelial cells, neutrophils, NK cells, TH17 cells, and B cells	Chemoattracts dendritic cells, effector/memory T lymphocytes, and naive B cells, elevated levels in atherosclerotic lesions [25,26]
CCL21	—	Changes in T-cell and macrophage responses due to dysfunction, reducing plaque stability, contributes to atherosclerosis [24]
CCL24	Activated immune cells (especially M2-type macrophages) and epithelial cells	Involved in inflammatory response, fibrosis process, and CVD attack [27,28].
CXCL1	Neutrophils, macrophages, epithelial cells	Promotes recruitment of neutrophils and monocytes/macrophages, induces myocardial infarction, atherosclerosis, and participates in organ fibrosis [29]
CXCL4/CXCL4L1/CXCL7	Platelet alpha granules	Involved in vascular injury inflammation, may act as an inflammatory mediator for vascular injury, promotes atherosclerosis [32,33]
CXCL8	—	Enhances chemotaxis and mitogenic effects on neutrophils, T cells, and vascular smooth muscle cells [10]
CXCL10	Endothelial cells, fibroblasts, keratinocytes, monocytes, T lymphocytes, macrophages	Enhances chemotaxis and mitogenic effects on neutrophils, T cells, and vascular smooth muscle cells [10]
CXCL12	Endothelial cells	Involved in the migration and differentiation of leukocytes, hematopoietic stem cells, and progenitor cells, plays a role in vascular inflammation [35]
CXCL16	Activated T cells, natural killer T cells	Promotes atherosclerosis [36,37]
CX3CL1	Endothelial cells, smooth muscle cells, macrophages, platelets	Participates in the development of inflammatory pathology such as atherosclerosis, mediates smooth muscle cell migration, has dual functions of chemotaxis and adhesion [20,38]
**Chemokine receptors**		
CCR1	Monocytes, neutrophils, T cells	Involved in inflammatory responses, promotes leukocyte recruitment, acts on chemokines CCL5, CCL3, CCL4 [39,40]
CCR2	—	Promotes neutrophil recruitment, may be involved in inflammatory responses [41,42]
CCR5	Monocytes, neutrophils, T cells	Involved in the activity of chemokines CCL3, CCL4, CCL5, atherosclerosis, regulates inflammatory infiltration [21,43]
CCR7	Lymphocytes, dendritic cells	Plays a role in the homing of lymphocytes and dendritic cells to secondary lymphoid tissues, regulates the migration of dendritic cell exosomes, helps improve cardiac function after myocardial infarction [44,45]
CCR8	CD4+αβ T cells, γδ T cells, NK cells	Recruits vascular Treg cells [46]
CXCR1, CXCR2	Leukocytes, fibroblasts, endothelial cells, smooth muscle cells	Participates in the mobilization of neutrophils and the early formation of atherosclerosis [47]
CXCR3	Immune cells, cardiovascular system cells	Regulates effector cell direction to infection sites during immune processes, associated with systemic diseases such as atherosclerosis, chronic Chagas cardiomyopathy, and hypertension [48,49]
CXCR4	Stem cells, peripheral blood leukocytes, endothelial cells, smooth muscle cells, myocardial cells	Coordinates rapid vascular reconstruction of damaged, ischemic, and regenerative tissues, plays a key role in ischemic responses [51]
CXCR6	—	Plays a pro-atherosclerotic role, regulates the production of pro-inflammatory IL-17A and T cell recruitment to atherosclerotic aortas [36]
CXCR7	—	Plays an important role in cardiovascular development, involved in the morphogenesis and remodeling of cardiac valves after myocardial infarction [51]
CX3CR1	Vascular smooth muscle cells, platelet-monocyte complexes	Guides cells to closely adhere to CX3CL1 under *ex vivo* flow conditions, involved in the formation of platelet-monocyte complexes [20,38]

## 3 Autophagy

### 3.1 Definition of autophagy

The term “autophagy” was coined by Christian de Duve, who discovered lysosomes. Autophagy is an essential intracellular degradation mechanism whose primary purpose is to eliminate proteins and damaged organelles caused by misfolding, thereby promoting cell survival under conditions of starvation and other stresses [4]. This mechanism is currently unique to eukaryotic organisms. Autophagy activity is relatively low under physiological conditions, is used to maintain homeostasis within the cell, and is activated to ensure normal functioning under complex and variable pathological conditions. Autophagy can take several forms, such as macroautophagy, microautophagy, and chaperone-mediated autophagy, with macroautophagy being the most extensively studied [52] and henceforth, referred to in this review simply as autophagy.

### 3.2 Autophagy signaling pathways

The pathways activated by autophagy include the mammalian target of rapamycin (mTOR)-dependent pathway and the mTOR-independent pathway. The mTOR-independent pathways involve various mechanisms, such as AMP-activated protein kinase (AMPK), mitogen-activated protein kinase (MAPK), phosphatidylinositol 3-kinase (PI3K), protein kinase A (PKA), phosphatase and tensin homolog (PTEN), and tumor protein p53 (p53) [4].

#### 3.2.1 mTOR-dependent pathway

mTOR exists in two distinct multiprotein complexes: mTORC1 and mTORC2. mTORC1 is composed of mTOR, RAPTOR, MLST8, PRAS40, and DEPTOR, and it is markedly sensitive to the drug rapamycin [53]. mTORC1 maintains autophagy at a basal level by binding to and phosphorylating the autophagy kinase complex ULK1/2, inhibiting the formation of autophagosomes. When mTORC1 is inactivated, it dissociates from the ULK1/2 complex, inducing autophagy, and ULK1 can activate the VPS34 complex by phosphorylating Beclin 1 to induce autophagy [54]. Although mTORC2 is less sensitive to rapamycin, its function can also be indirectly inhibited by long-term treatment with rapamycin, which contributes to the regulation of autophagy [53].

#### 3.2.2 mTOR-independent pathways

**Figure 1:**
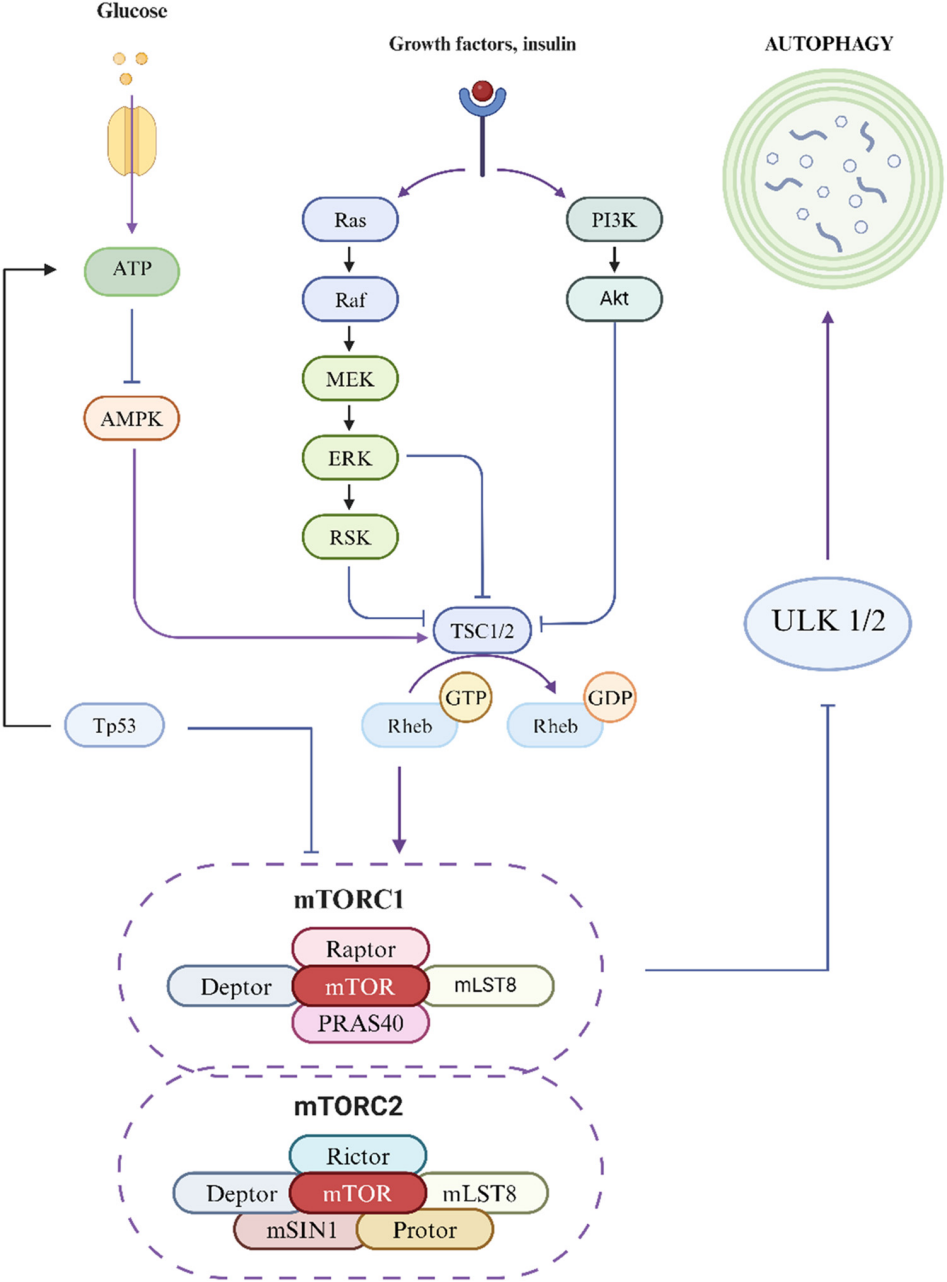
Autophagy signaling pathway diagram.

When there is insufficient energy within the cell, AMPK is activated and stimulates autophagy. AMPK directly promotes autophagy by phosphorylating ULK1, thereby inhibiting the binding of mTORC1 and enhancing the turnover of autophagy after glucose deprivation. It can also directly promote autophagy by phosphorylating autophagy-related proteins in the mTORC1, PIK3C3/VPS34 complex, or indirectly promote autophagy by regulating the expression of downstream autophagy-related genes through transcription factors such as FOXO3, TFEB, and BRD4 [55]. The RAS/RAF/MEK/ERK signaling pathway is one of the most important pathways in the MAPK system and is involved primarily in regulating cell proliferation, differentiation, and apoptosis. This pathway begins with the RAS protein, a monomeric small GTPase encoded by the NRAS, HRAS, and KRAS genes. Upon activation by growth factors or other signaling molecules, RAS is activated to form a complex of Src homology collagen (SHC)/growth factor receptor-bound protein 2 (GRB2)/son of sevenless (SOS), which stimulates SOS to replace GTP with RAS, generating active RAS-GTP. Activated RAS recruits RAF kinase to the plasma membrane, and the activation of RAF requires specific phosphorylation and dephosphorylation processes. RAF kinase phosphorylates downstream MEK1/2 and forms a tetramer composed of RAF-MEK dimers, further promoting the activation of MEK. Ultimately, ERK1/2, the terminal kinase of the cascade reaction, is phosphorylated and activated by MEK1/2 and then translocates to the cell nucleus or remains in the cytoplasm, where it regulates a variety of substrates [56]. PI3K, a member of the phosphoinositide kinase family, possesses both PI3k activity and serine/threonine kinase activity. It is capable of phosphorylating PI to produce phosphatidylinositol 3-phosphate (PI3P). Specifically, class III PI3K (PI3KC3) can phosphorylate PI to generate PI3P, which functions downstream of the ULK complex as an important protein that regulates autophagy. PI3KC3 has two complexes: PI3KC3-C1 and PI3KC3-C2, with PI3KC3-C1 acting in the early stages of autophagy and PI3KC3-C2 acting mainly in the later stages [57]. The PTEN protein is a negative regulator of the PI3K pathway and has proautophagy activity. PKA is a negative regulator of autophagy that mainly acts on Atg1, Atg8, and Atg13 [58]. The Tp53 gene encodes the transcription factor p53, which is involved in DNA repair, aging, cell cycle control, autophagy, and apoptosis and can regulate various targeted genes to activate AMPK or inhibit mTOR, promoting autophagy [59] (Figure 1).

mTOR exists in two distinct multiprotein complexes: mTORC1 and mTORC2. mTORC1 is composed of mTOR, Raptor, mLST8, PRAS40, and Deptor. It is highly sensitive to rapamycin, a known inhibitor. mTORC1 inhibits autophagy by binding to and phosphorylating the autophagy kinase complex ULK1/2, maintaining autophagy at a baseline level. Inactivation of mTORC1 leads to the dissociation of the ULK 1/2 complex, triggering autophagy. mTORC2 consists of mTOR, Rictor, mLST8, mMSIN1, Protor, and Deptor. It is less sensitive to rapamycin, but its function can be indirectly inhibited by long-term treatment with the drug, thus participating in the regulation of autophagy. When cellular energy is scarce, Tp53 activates AMPK, which inhibits the binding of mTORC1 and promotes autophagy. Upon activation by growth factors or other signaling molecules, the RAS protein is first activated, promoting the translocation of RAF kinase to the cell membrane, which triggers a series of phosphorylation events, successively activating MEK and ERK. Ultimately, the ERK proteins are translocated to the cell nucleus or function within the cytoplasm. Concurrently, PI3K phosphorylates phospholipids to generate PI3P, promoting the activation of Akt, which in turn inhibits TSC1/2 and Rheb, promoting autophagy.

## 4 Research progress on the impact of chemokines on autophagy in CVD

Research on how chemokines affect autophagy and thereby alleviate CVD is increasing. To date, chemokines such as CXCR4/CXCL12, CXCL16/CXCR6, and CX3CL1 have been found to protect the heart by inducing autophagy when inhibited.

CXCR4 is one of the specific receptors for CXCL12, and its activation upon binding with CXCL12 plays a role in signal transduction related to inflammation, chemotaxis, survival, and proliferation, with high expression of CXCR4 being closely related to the stability of atherosclerosis [60]. Research has shown that CXCL12-CXCR4 can regulate blood lipids, activate vascular inflammatory responses, promote tumor endothelial proliferation, stimulate angiogenesis, and exacerbate insulin resistance. Autophagy plays a key role in the biological functions of vascular endothelial cells, and its regulation through the PI3K/AKT/mTOR pathway is also crucial for autophagy. Studies have shown that after the specific knockout (KO) of CXCR4, the activation of the PI3K/AKT/mTOR signaling pathway is inhibited. This leads to an increase in the expression of the autophagy-related proteins LC3 and Beclin1 and a decrease in the level of the autophagy substrate p62, indicating increased autophagy activity. Further experimental results have indicated that blocking CXCR4 can promote autophagy in macrophages through the PI3K/AKT/mTOR pathway. This mechanism can not only reduce the degree of atherosclerosis but also improve myocardial structure, thereby reducing the severity of coronary artery disease. These findings reveal the important role of the CXCL12-CXCR4 signaling pathway in CVD and provide potential targets for the development of new therapeutic strategies. By regulating this pathway, especially by promoting macrophage autophagy, new directions may be provided for the prevention and treatment of atherosclerosis and related CVD [7]. Another study showed that blocking TLR2/CXCR4 can significantly delay or perhaps even reverse atherosclerosis induced by Chlamydia pneumoniae infection [61].

CXCL16 belongs to the CXC chemokine family and exists in both membrane-bound and soluble forms, with soluble CXCL16 in the serum having a proatherosclerotic effect. CXCR6 is the specific receptor for CXCL16, and the CXCL16/CXCR6 axis plays an important role in pathological mechanisms following I/R injury in cardiac remodeling and heart failure development. IFN-γ plays a crucial role in immune regulation, as it can modulate immune defense mechanisms by activating autophagosomes. These autophagosomes are essential for inducing the death of activated T lymphocytes and macrophages. Although autophagy induced by IFN-γ may help alleviate excessive inflammatory responses during I/R injury in some cases, an increasing number of studies suggest that autophagy may actually have detrimental effects on ischemic heart disease during the reperfusion phase. In one study, a CXCR6 KO mouse model was used to induce myocardial injury through myocardial I/R surgery and to assess myocardial function and infarct size. The results showed that after 6 h of reperfusion, the serum IFN-γ level significantly increased, accompanied by an increase in the level of the autophagy-related protein Beclin1. Notably, CXCR6-deficient mice presented smaller myocardial infarct areas and better cardiac function than control mice did, indicating that CXCR6 deficiency inhibited monocyte infiltration and IFN-γ production, thereby alleviating myocardial I/R injury. The mechanism may be that I/R triggers the infiltration of monocytes into the myocardium and activates IFN-γ-dependent autophagy signaling pathways through CXCL16/CXCR6. Therefore, disrupting the CXCL16/CXCR6 signaling cascade has a cardioprotective effect on I/R injury. These findings suggest that the absence of CXCR6 may protect the heart from severe damage during reperfusion by blocking the secretion of IFN-γ and thereby inhibiting IFN-γ-dependent autophagy. This mechanism provides new insights into the role of CXCR6 in I/R injury and opens new directions for potential therapeutic strategies [8].

**Figure 2:**
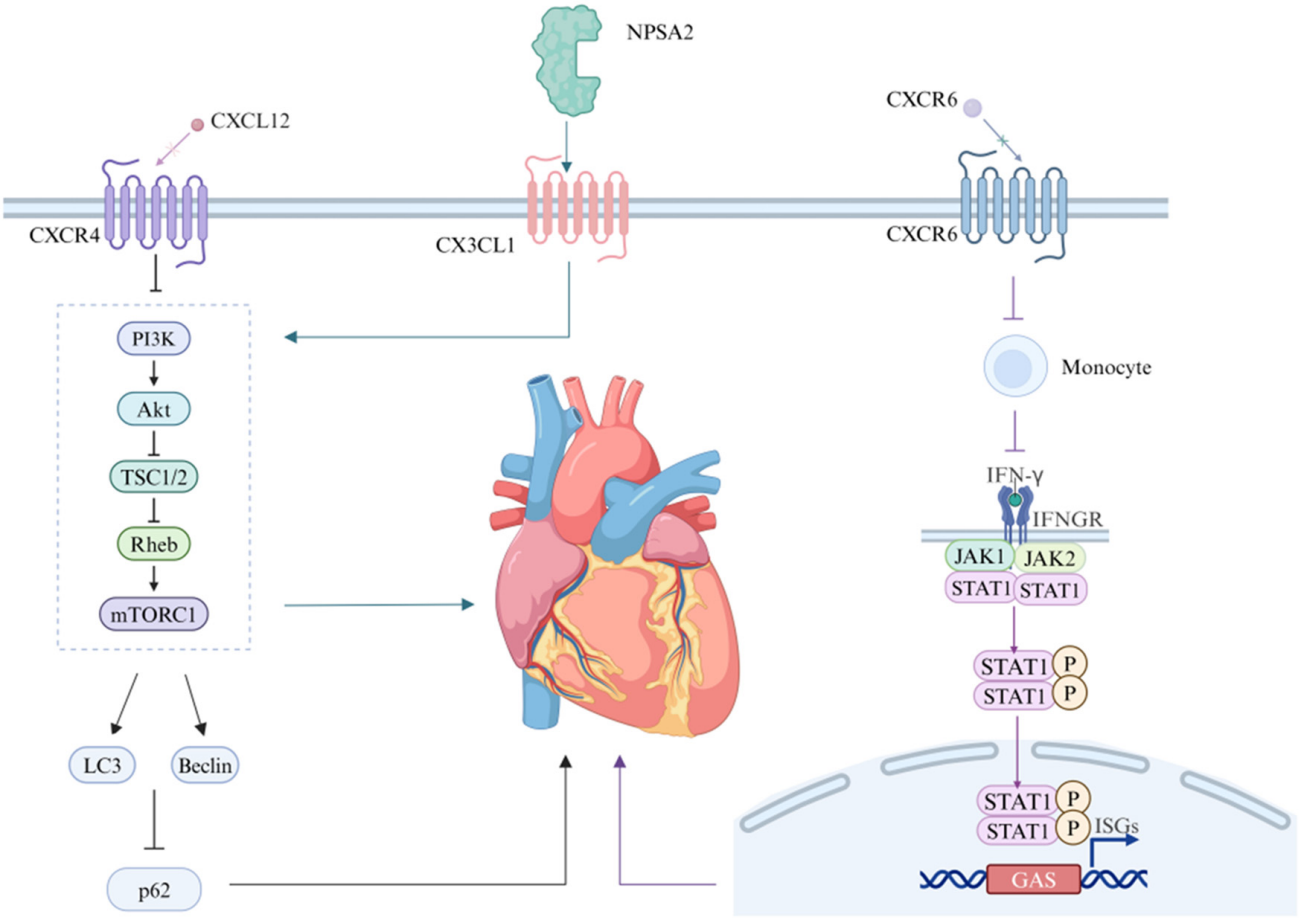
Chemokines regulate autophagy to improve cardiac injury.

CX3CL1 is a unique membrane-bound chemokine with both chemoattractant and adhesive functions. The serum level of CX3CL1 is associated with CVD, such as carotid artery stenosis, unstable angina, and systolic heart failure. NPAS2 has been demonstrated to regulate the autophagy process through the CX3CL1/AKT pathway, with high expression levels of NPAS2 capable of inhibiting autophagy and apoptosis in cardiomyocytes. Studies have indicated that AKT/mTOR-mediated autophagy is closely associated with myocardial I/R injury. However, other studies have shown that Beclin1-mediated autophagy is also involved in the process of myocardial I/R injury. The inhibition of Beclin1 expression in cardiomyocytes can reduce the degree of autophagy and apoptosis induced by I/R. The primary mechanism involves the upregulation of Beclin1 induced by reactive oxygen species (ROS), leading to defective autophagosome maturation and increased cell death. Experimental results have revealed that NPAS2 can promote the transcription of CX3CL1. In H9C2 cells, NPAS2 binds to the promoter region of CX3CL1. NPAS2 overexpression promotes the expression of CX3CL1, whereas NPAS2 suppression reduces the expression of CX3CL1. CX3CL1 has been confirmed as a direct transcriptional target of NPAS2 in both breast cancer and cardiomyocytes. NPAS2 overexpression increases the level of CX3CL1, thereby promoting the phosphorylation of AKT and mTOR and participating in the regulation of autophagy. Notably, the inhibition of CX3CL1 can reverse the protective effect of NPAS2 on myocardial injury, indicating that the NPAS2/CX3CL1 pathway plays a significant role in myocardial protection. This discovery provides a new potential therapeutic target for myocardial protection [9] (Figure 2) (Table 2).

**Table 2:** Chemokines that protect the heart by modulating autophagy

Chemokine ligand	Chemokine receptor	Autophagy regulation role	Related research/mechanism of action
CXCR4	CXCL12	Regulates autophagy through the PI3K/AKT/mTOR pathway, alleviating atherosclerosis, and improving myocardial structure.	Blocking CXCR4 can promote autophagy in macrophages, alleviate atherosclerosis, improve myocardial structure, reduce the severity of coronary artery disease, and reverse atherosclerosis induced by Chlamydia pneumoniae infection by blocking TLR2/CXCR4 [7].
CXCL16	CXCR6	Has a pro-atherosclerotic effect; CXCR6 deficiency inhibits monocyte infiltration and IFN-γ production, reducing myocardial I/R injury	CXCR6 deficiency reduces monocyte infiltration and IFN-γ production, mitigating myocardial I/R injury, possibly through CXCL16/CXCR6-dependent paracrine IFN-γ inducing cardiac autophagy [8].
CX3CL1	CX3CR1	Has dual functions of chemotaxis and adhesion; NPAS2/CX3CL1 is involved in the regulation of autophagy.	NPAS2 promotes the transcription of CX3CL1, and the NPAS2/CX3CL1 axis is involved in the regulation of autophagy by promoting the phosphorylation of AKT and mTOR, playing a role in myocardial protection [9].

Blockade of CXCR4 can inhibit the activation of CXCR4 after binding with CXCL12 and regulate autophagy through the PI3K/AKT/mTOR pathway to improve and reduce atherosclerosis and improve cardiac structure. CXCR6 deficiency can inhibit monocyte infiltration and the production of IFN-γ, thereby reducing myocardial I/R injury. NPAS2 promotes the transcription of CX3CL1 and is involved in the regulation of autophagy to protect the heart.

## 5 Conclusion and expectations

Autophagy is crucial for maintaining cellular homeostasis and plays a vital role in cardiomyocytes under both basal and stress conditions, helping to sustain cardiac function. Chemokines, a group of inflammatory mediators rapidly released upon platelet activation, also play a significant role in the development of CVD such as atherosclerosis. Therefore, modulating chemokines to regulate autophagy may have an impact on CVD. For example, the binding of CXCR4 to CXCL12 promotes macrophage autophagy through the PI3K/AKT/mTOR pathway, which alleviates atherosclerosis and improves myocardial structure. Additionally, blocking TLR2/CXCR4 can significantly delay or even nearly reverse atherosclerosis induced by Chlamydia pneumoniae infection. Soluble CXCL16 in the serum, when bound to CXCR6, plays an important role in pathological mechanisms following I/R injury in cardiac remodeling and heart failure development. CXCR6 deficiency activates the IFN-γ-dependent autophagy signaling pathway, inhibiting monocyte infiltration and mitigating myocardial I/R injury. CX3CL1, with its chemotactic and adhesive functions, is upregulated by NPAS2, which promotes the phosphorylation of AKT and mTOR and is involved in the regulation of autophagy. The NPAS2/CX3CL1 axis plays a role in myocardial protection, and the therapeutic effect of NPAS2 on myocardial injury can be reversed by inhibiting CX3CL1.

Research has shown the feasibility of using chemokines to regulate autophagy for cardiac protection. Therefore, in addition to the known chemokines CXCR4, CXCL12, CXCR6, and CXCL16, it is also important to investigate the regulatory mechanisms and mechanisms of action of other chemokines associated with CVD to verify whether they can also prevent or treat CVD by regulating autophagy. Furthermore, on the basis of the interaction between chemokines and the autophagy pathway, new molecular targets can be identified, and targeted drugs can be developed to provide more effective treatment plans. For example, drugs that can specifically block or activate chemokine receptors to regulate autophagy and improve the prognosis of CVD patients may be designed. By detecting the levels of chemokines and their receptors in the blood, early diagnostic tools can be developed to predict the risk of CVD, achieve early intervention and treatment, and improve patient survival rates and quality of life. Large-scale clinical trials should be conducted to ensure the actual effectiveness and safety of the regulation of autophagy by chemokines in the treatment of CVD and to determine the best treatment plans and medication strategies.

In summary, in-depth research on the interaction between chemokines and autophagy in CVD will not only help reveal the mechanisms of diseases but also provide new ideas and strategies for the diagnosis and treatment of CVD. It is also important to explore whether chemokines can contribute to the primary prevention of CVD. Such research will promote the innovation and application of related treatment methods and ultimately improve the health and well-being of patients.
